# Clinical study and the diagnosis of lumpy skin disease in cattle using genomic, immunological, and pathological indicators

**DOI:** 10.3389/fvets.2026.1818746

**Published:** 2026-04-23

**Authors:** Haifa Ali Alqhtani, Mohamed Marzok, Ahmed A. Elsayed, Ahmed H. Ghonaim, Adel I. Almubarak, Isam Mohamed ElJalii, Hussein Babiker, Ahmed Omer Alameen, Moustafa Shoukry, Sherief M. Abdel-Raheem, Ibrahim M. El-Sabagh, Mohamed F. Hamed, Fatmah Ahmed Safhi, Elham Mohammed Alzahrani, Ahmed Ateya, Reham Karam

**Affiliations:** 1 Department of Biology, College of Science, Princess Nourah bint Abdulrahman University, Riyadh, Saudi Arabia; 2 Department of Clinical Studies, College of Veterinary Medicine, King Faisal University, Al-Ahsa, Saudi Arabia; 3 Department of Animal Health and Poultry, Animal and Poultry Production Division, Desert Research Center (DRC), Cairo, Egypt; 4 National Key Laboratory of Agricultural Microbiology and Hubei Hongshan Laboratory, Huazhong Agricultural University, Wuhan, China; 5 Department of Biomedical Sciences, Faculty of Veterinary Medicine, King Faisal University, Al-Ahsa, Saudi Arabia; 6 Department of Public Health, College of Veterinary Medicine, King Faisal University, Al-Ahsa, Saudi Arabia; 7 Central Biotechnology Laboratory, College of Veterinary Medicine, King Faisal University, Al-Ahsa, Saudi Arabia; 8 Department of Pathology, Faculty of Veterinary Medicine, Mansoura University, Mansoura, Egypt; 9 Department of Development of Animal Wealth, Faculty of Veterinary Medicine, Mansoura University, Mansoura, Egypt; 10 Department of Virology, Faculty of Veterinary Medicine, Mansoura University, Mansoura, Egypt; 11 Weqaa Central Laboratory, Weqaa Center, Ministry of Environment, Water and Agriculture, Riyadh, Saudi Arabia

**Keywords:** cattle, genomic, immunity, indicators, lumpy skin disease, pathology

## Abstract

**Introduction:**

Lumpy skin disease (LSD) is a transboundary animal disease that has serious implications for livestock trade and food security. This study aimed to investigate the molecular and immunological determinants of LSD in vaccinated cattle that developed clinical disease (vaccine breakthrough cases), and to identify potential biomarkers to support disease surveillance and control strategies.

**Methods:**

Blood samples were collected from 50 clinically healthy cattle (controls) and 50 vaccinated cattle with clinical signs of LSD. Each blood sample was divided into two portions: EDTA-treated blood was used for viral DNA and total RNA extraction for assessing gene expression of immune and antioxidant genes, PCR amplification, partial sequencing, and phylogenetic analysis of the ORF95 gene, whereas serum samples were used to evaluate biochemical, antioxidant, and immunological parameters. In addition, 2–3 skin nodules were surgically excised from the affected animals for pathological examination. Gene expression and sequencing analyses of TLR7, TLR8, TLR9, SOD3, CAT, and GPX were performed in both groups, and single nucleotide polymorphisms (SNPs) were detected.

**Results and discussion:**

Clinically affected cattle exhibit pyrexia (40–41 °C) and characteristic nodular skin lesions. Partial ORF95 sequences obtained from infected cattle clustered closely with the circulating field strains, indicating active field virus circulation despite vaccination. Histopathological examination revealed typical lesions including epidermal necrosis with eosinophilic intracytoplasmic inclusions, dermal ulceration, and fibrinous exudation. Serum analyses demonstrated significant alterations in inflammatory cytokine levels, oxidative stress indices, and metabolic parameters, consistent with systemic inflammation and oxidative imbalance. Molecular analyses showed significant upregulation of toll-like receptor 7 (TLR7), toll-like recep tor 8 (TLR8), and toll-like receptor 9 (TLR9), accompanied by the downregulation of antioxidant genes; superoxide dismutase 3 (SOD3), catalase (CAT), and glutathione peroxidase (GPX) in infected cattle (*p* < 0.05). Five SNPs were identified in the investigated genes in the affected animals. Overall, these findings indicate a pronounced inflammatory and oxidative stress response in LSDV-infected cattle, and suggest the involvement of specific genetic variants that may contribute to disease susceptibility.

## 1 Introduction

Lumpy skin disease (LSD), caused by the lumpy skin disease virus (LSDV), belongs to the genus Capripoxvirus within the family *Poxviridae* (1). LSD is a highly contagious, emerging, and critically notifiable transboundary animal disease that severely affects cattle and water buffalo, leading to substantial economic losses (2). Owing to its transboundary nature and significant impact on livestock productivity and trade, LSD poses a serious threat to global food security (3). In addition, recent reviews highlight the rapid geographic expansion of LSD virus (LSDV) and emphasize existing gaps in understanding host susceptibility, vaccine efficacy, and viral evolution (4), underscoring the urgent need for further molecular and immunological studies to support effective disease control.

The recent emergence of LSD in Europe, with outbreaks reported in France and Italy from June to July 2025, provides compelling evidence of its capacity for transboundary spread and highlights the risk of incursion into regions that have remained free of the disease for decades (5). In 2017, the recombinant vaccine-like strain LSDV RUS-SIA/Saratov/2017 was first identified; since then, multiple recombinant strains have been reported. Whole-genome sequencing of these strains revealed distinct recombination events and single nucleotide polymorphisms (SNPs), leading to their provisional classification into five clusters, designated 2.1–2.5 (6).

LSDV shares ~96% antigenic similarity and exhibits serological cross-reactivity with Sheep pox and Goat pox viruses (7). LSDV is primarily transmitted through blood-feeding arthropods such as stable flies and ticks (8). Less frequently, direct contact with skin lesions, saliva, nasal discharge, milk, or semen from infected animals can transmit the virus (9). Clinical manifestations range from acute to subacute and chronic, with mild to severe symptoms including fever, nasal discharge, lacrimation, hypersalivation, anorexia, lethargy, and characteristic nodular lesions on the skin and mucous membranes (10, 11).

Although average morbidity rates typically range from 1 to 5%, localized outbreaks may result in morbidity rates as high as 80–90% and mortality rates reaching 40% or higher in severe cases (12–14). The economic burden of LSD includes decreased productivity, damaged hides, medical and preventive expenses, as well as miscarriage and sterility in males and females, respectively (12). LSDV can be definitively detected using a mix of laboratory methods, including electron microscopy, serology, molecular tests, and viral isolation (15, 16). A thorough evaluation of various facets of the disease is necessary to improve our understanding of its pathogenesis, diagnosis, and control strategies through effective preventive and therapeutic measures, especially considering the recent rapid spread of LSD into uninitiated areas and its severe economic consequences (17). LSDV is genetically stable and is controlled by live attenuated vaccines based on either sheep poxvirus or neethling-based LSDV vaccines (18). A recombinant strain emerged in Asia only in 2017. This strain was found to harbor genetic sequences from known vaccine strains from Clades 1.1 (Neethling strain) and 1.2 (KSGP strain), and possibly even Goat pox virus sequences (19).

According to Sharma et al. (20), oxidative stress frequently occurs when there is a decrease in antioxidants and an abundance of oxidants such as free radicals or reactive oxygen species. However, through defense mechanisms, animal bodies may control the excess production of free radicals (21). Catalases and other antioxidant enzymes are crucial for these processes (22–24). Reactive oxygen species (ROS) and reactive nitrogen species (RNS) can be created in enormous quantities during the immunological response to infection, which damages tissues oxidatively (25, 26).

Cytokines regulate several cellular and physiological processes, and play crucial roles in the immune system of dairy cows. Furthermore, they function as messenger cells in immune and inflammatory responses (27, 28). The animal’s immune system, level of immunization, type of virus, and frequency of vectors in the surrounding environment influence how an LSD infection turns out (12). Understanding the genetic correlates of immune response is crucial for identifying animals with inherent resistance or tolerance to infectious agents. However, studying the genetic basis of disease resistance in livestock poses several challenges, including low heritability, infrequent natural exposure events, ethical constraints, and unfavorable genetic correlations with production traits (29). Toll-like receptors (TLRs) are involved in the innate immune response that recognizes the molecular signatures of pathogens, known as pathogen-associated molecular patterns (PAMPs). Among the TLR family, four members are specialized in detecting virus-specific PAMPs namely TLR3, 7, 8, and 9 (30).

However, the genetic foundation of the immune response can be clearly recognized by the occurrence of a spontaneous epidemic and nearly uniform exposure of animals to infectious pathogens. It is unclear how the immune system defends itself against LSD infection in natural hosts. Developing precise disease management strategies and potent vaccinations requires a deep understanding of the genetic pathways underlying the best possible immune response to pathogenic agents. It is critical to understand the genetic underpinnings of variation in the immune response observed in cow herds. In-depth knowledge of the host immune response to LSD infection, such as cytokines linked to inflammatory processes, may prove useful in developing biomarkers to distinguish between infected animals and those that are tolerant or resilient.

Despite the global importance of the disease, limited data are available regarding the clinicopathological and molecular features of naturally occurring LSDV infections, particularly in endemic regions such as Egypt. Therefore, this study aimed to clinically and diagnostically investigate lumpy skin disease in cattle using integrated genomic, immunological, and pathological indicators. This integrated investigation seeks to advance our understanding of host-pathogen interactions in LSD and identify potential biomarkers of disease susceptibility and resilience.

## 2 Materials and methods

### 2.1 Animals

A total of 100 adult Holstein–Friesian female cows (age: 6–7 years; mean ± SD: 6.3 ± 0.3; body weight: 625–700 kg; mean ± SD: 669.1 ± 21.9) were included in this study. All animals were selected from the same private farm in the El Salam Canal area to minimize environmental and management-related variability. Cows were maintained under identical housing, feeding, and management conditions and had no prior history of metabolic or infectious diseases. All animals had been previously vaccinated against lumpy skin disease using a live attenuated Neethling strain vaccine (Lumpyvax®, MSD Animal Health), batch number: A12345, as recorded in the farm vaccination log. The vaccine was administered as a single standard dose (as recommended by the manufacturer) via subcutaneous injection prior to the outbreak. Sampling was conducted during an LSD outbreak between August and October 2023. Based on clinical examination and molecular testing, animals were classified into two groups: (1) 50 vaccinated cattle exhibiting clinical signs consistent with LSD (affected group), and (2) 50 vaccinated cattle that remained clinically healthy (control group). Clinical examination included assessment of rectal temperature, heart rate, respiratory rate, and ruminal contractions. To confirm group classification, all control animals tested negative for lumpy skin disease virus (LSDV) by PCR and showed no clinical signs of infection. Affected animals exhibited typical clinical manifestations, including firm, circumscribed skin nodules distributed over the head, neck, trunk, perineum, udder, teats, subcutaneous tissue, and muscles with ulceration and necrosis, accompanied by systemic signs such as fever, depression, inappetence, reduced milk yield, respiratory and ocular complications, and in severe cases, recumbency.

### 2.2 Blood sampling

Sterile vacuum blood collection tubes (Vacutainer®; Becton Dickinson) were used for sample collection. For hematological analysis, K2-EDTA tubes (purple-top, 4 mL) were used to prevent coagulation, whereas for serum biochemical and immunological assays, plain serum tubes (red-top, 5 mL) were used. All tubes were handled in accordance with the manufacturer’s instructions to ensure sample integrity. All samples were immediately transported on crushed ice to the laboratory for further processing. Tubes containing whole blood were used for DNA extraction of virus and RNA extraction for investigating gene expression profile of immune and antioxidant indicators, whereas those in plain tubes were kept overnight at 4 °C and centrifuged at 3,000 rpm for 15 min. Only clear sera were collected, aliquoted, and kept frozen at −20 °C for subsequent biochemical analyses.

### 2.3 Virological examination

The 10 clinical samples were intentionally selected from animals showing typical and severe clinical manifestations and the presence of lumpy skin disease to ensure a high viral load and reliable PCR detection. Five other samples from apparently healthy animals were randomly selected and tested. This targeted selection was performed to confirm the presence of LSDV and to obtain representative sequences for molecular characterization, rather than to estimate the prevalence. Genomic DNA was extracted from whole blood using the QIAamp® DNA Blood Kit following the manufacturer’s protocol. Briefly, blood samples were lysed using proprietary lysis buffer containing proteinase K to disrupt cell membranes and digest proteins. The released DNA was selectively bound to a silica-based membrane in a spin column under high-salt conditions. After several washing steps to remove contaminants and inhibitors, the purified DNA was eluted in a low-salt buffer and stored at −20 °C until further PCR analysis. PCR was performed to partially amplify the LSDV core protein-producing gene (ORF095). Cycling procedures were performed according to the Emerald Amp GT PCR Master Mix (Takara) Code No. RR310 according to the manufacturer’s instructions using the primers 5ʹATGGACTTCATGAAAAAATATACT3` and 5ʹTTTGCTGTTATTATCATCCAG3` (31). Initial polymerase enzyme activation was performed at 94 °C for 5 min, followed, 40 cycles of denaturation at 94 °C for 30 s. Primer annealing at 42 °C for 50 s followed by extension at 72 °C for 45 s. final extension was performed at 72 °C for 10 min. A positive control (internal positive control) and negative extraction control were added to each PCR run. The amplified DNA migrated in an electric field to produce sharp bands compared to the DNA Ladder. One clear band was excised and sent for sequencing at the Animal Health Research Institute (AHRI, Dokki, Giza, Egypt). Phylogenetic analysis was performed on DNA sequences generated using the neighbor-joining tree in MEGA 11 software (32).

### 2.4 Histopathological examination

Skin lesion biopsies were collected aseptically using a sterile punch or scalpel and immediately fixed in 10% neutral buffered formalin. The samples were then processed using routine histopathological procedures, including dehydration through graded alcohol solutions, clearing in xylene, embedding in paraffin, sectioning at 4–5 μm, and staining with hematoxylin and eosin for microscopic examination (33).

### 2.5 Biochemical analysis

Serum concentrations of biochemical, oxidative stress, cytokine, and immunological parameters from the same animals included in the study, with *n* = 50 LSDV-infected cattle and *n* = 50 control cattle for each parameter, were measured using the respective commercial kits according to the manufacturer’s instructions. Biochemical parameters, including total protein, albumin, glucose, cholesterol, urea, creatinine (Gamma Trade Company, Egypt), calcium, phosphorus, electrolytes, and liver and muscle enzymes (Spectrum Company, Egypt), were quantified using a selective chemistry analyzer (Apple 302, USA). Oxidative stress markers (MDA, CAT, GPx, GSH) were measured spectrophotometrically using (Biodiagnostic Egypt) kits. Cytokines IL-6 (BOSTER BI-OLOG-ICAL TECHNOLOGY, CAT No: EK0412), IL-10 (ELISA kits from Ray Biotech Company®), TNF-*α* (AVIVA SYSTEM BIOLOGY), and immunoglobulins IgG (Cell Sciences company, CAT No: CKR004A), IgA (EAGLE BIOSCIENCE company), and IgM (Genemed Synthesis, CAT. NO EK-6480) were quantified using ELISA kits, whereas α-, *β*-, and *γ*-globulin concentrations were determined using commercial radial immuno-diffusion plates.

### 2.6 Gene expression profile

Total RNA was extracted from cattle (*n* = 50 LSD and *n* = 50 healthy) using TRIzol reagent according to the manufacturer’s instructions (RNeasy Mini Ki, Catalogue No. 74104) for assessing expression of immune and antioxidant genes. The amount of extracted RNA was quantified using a NanoDrop® ND-1000 Spectrophotometer. Total RNA was isolated from freshly obtained blood samples using TRIzol reagent in combination with the Directzol RNA MiniPrep extraction kit, in accordance with the manufacturer’s protocol. Briefly, 300 μL of the TRI reagent was mixed thoroughly with 100 μL of fresh blood and incubated for 5 min to allow complete lysis. The lysate was then centrifuged at 10,000–16,000×*g* for 1 min to pellet the insoluble debris, after which the clear supernatant was carefully transferred into an RNase-free tube. An equal volume of 95–100% ethanol was added to the supernatant and mixed gently to facilitate RNA binding. The resulting solution was loaded onto a Zymo-Spin column, placed in a collection tube, and centrifuged at 10,000–16,000×*g* for 30 s. The flow-through was dis-carded, and the column was returned to the collection tube. Subsequently, 400 μL of Directzol RNA PreWash buffer was applied to the column and centrifuged; this washing step was performed twice. The column was then washed with 700 μL of RNA wash buffer and centrifuged for 2 min to remove any residual wash solution. After washing, the column was carefully transferred into a clean RNase-free tube. RNA was eluted by adding 50 μL of DNase/RNase-free water directly to the column matrix, followed by centrifugation.

The cDNA of each sample was synthesized from the extracted RNA according to the manufacturer’s protocol (Thermo Fisher, Catalog no, EP0441). The gene expression patterns for coding fragments of genes encoding immune (TLR7, TLR8, and TLR9) and antioxidants (SOD3, CAT, and GPX) were assessed by quantitative RT-PCR using SYBR Green PCR Master Mix (2x SensiFastTM SYBR, Bioline, CAT No: Bio-98002). Relative quantification of mRNA levels was performed by real-time PCR using SYBR Green PCR Master Mix (Quantitect SYBR Green PCR kit, Catalog no, 204141). Primer sequences were designed according to the PubMed published sequences of *Bos taurus* (Table 1). The housekeeping gene, GAPDH was used as a constitutive control for normalization. The reaction mixture was carried out in a total volume of 25 μL consisted of total RNA 3 μL, 4 μL 5x Trans Amp buffer, 0.25 μL reverse transcriptase, 0.5 μL of each primer, 12.5 μL 2x Quantitect SYBR green PCR master mix and 8.25 μL RNase free water. The final reaction mixture was placed in a thermal cycler and the following program was carried out: reverse transcription at 50 °C for 30 min, primary denaturation at 94 °C for 10 min followed by 40 cycles of 94 °C for 15 s, annealing temperatures for 1 min, as shown in Table 1, and 72 °C for 30 s. At the end of the amplification phase, melting curve analysis was performed to confirm the specificity of the PCR product. The relative expression of each gene per sample in comparison with GAPDH was calculated according to the 2^−ΔΔCt^ method (34).

**Table 1:** Oligonucleotide primers sequence, accession number, annealing temperature and PCR product size of immune and antioxidant genes used in real time PCR.

Gene	Primer	Product length (bp)	Annealing Temperature (°C)	Accession number
*TLR7*	F5′-CCAATGTGGACATTGAAGAGAC-3′ R5′-AGTCGAACAGGTACGCAGTTGA-3′	305	60	NM_001033761.1
*TLR8*	F5′-TTCTTCACTGAAGCCAGTTATC-3′ R5′-TGTTGTAACTGGTTGTCTTCCA-3′	338	58	NM_001033937.1
*TLR9*	F5′-TCTCTCCTGGTGCAGGCGGCG-3′ R5′-CAGGCACGGTCGTGATGCCGT-3′	385	58	KX138608.1
*SOD*	F5′-TCCTCGGGTCCCAGGTGCTCGA-3′ R5′-AGGCCGGAGCTGCCGGAAGAGC-3′	300	60	NM_001428313.1
*CAT*	F5′-GATCCAGCCAGCGACCAGATG-3′ R5′-GATTCTCCAGCAACAGTGGAGA-3′	344	58	NM_001035386.2
*GPX1*	F5′-GCGCACAGTGTACGCCTTCTC-3′ R5′-GGCGAAGAGCGGATGCGCCTTC-3′	343	60	NM_174076.3
*GAPDH*	F5′-GATGGTGAAGGTCGGAGTGAA-3′ R5′-GACGAGCTTCCCGTTCTCTG-3′	199	60	NM_001034034.2

TLR7, Toll like receptor 7; TLR8, Toll like receptor 8; TLR9, Toll like receptor 9; SOD3, Superoxide dismutase 3; CAT, Catalase; GPX1, Glutathione peroxidase 1; GAPDH, Gapdh glyceralde-hyde-3-phosphate dehydrogenase.

### 2.7 DNA sequencing and SNPs detection

To detect SNPs in genes investigated in control and LSD-affected cattle, PCR products with target amplification were sent for DNA sequencing in forward and re-verse directions using an ABI 3730XL DNA sequencer (Applied Biosystems, USA), depending on the enzymatic chain terminator technique developed by Sanger et al. (21). Single nucleotide polymorphisms (SNPs) were identified by aligning forward and reverse Sanger sequencing reads against reference gene sequences. Only high-quality base calls with clear chromatographic peaks were considered.

Analysis of DNA sequencing data was carried out using chromas 1.45 and BLAST 2.0 (35). Differences were classified as SNPs between the PCR products of the investigated genes and reference sequences available in GenBank.

Reference sequences were obtained from the NCBI GenBank database^1^ and included TLR7 (NM_001033761.1), TLR8 (NM_001033937.1), TLR9 (KX138608.1), SOD3 (NM_001428313.1), CAT (NM_001035386.2), and GPX1 (NM_174076.3). All sequences were accessed on February 18, 2025.” based on the data alignment of DNA sequencing, variation in the amino acid sequence of the investigated genes between the enrolled cows was performed using the MEGA 12 software package (36).

### 2.8 Statistical analysis

Statistical analyses were performed using IBM SPSS Statistics (version 23). An independent-samples t-test was used to compare the mean values between the LSD and healthy groups, and results are presented as mean ± SD. The sample size was determined using *a priori* power analysis for comparison of two independent groups. Assuming a moderate effect size (Cohen’s d = 0.6), a two-tailed significance level (*α*) of 0.05, and a statistical power of 80% (1 − *β* = 0.80), the minimum required sample size was calculated to be 45 animals per group. To account for potential data loss or sample exclusion, the sample size was increased to 50 animals per group (total *n* = 100).

Differences in SNP frequencies between LSD and healthy cows were assessed using the Chi-square test in SPSS (version 23). Linear Discriminant Analysis (LDA) was performed to evaluate whether gene-level SNP averages could discriminate between the two groups, using the six genes as predictors and health status as the grouping variable. Statistical significance was set at *p* ≤ 0.05.

## 3 Results

### 3.1 Clinical findings

**Figure 1:**
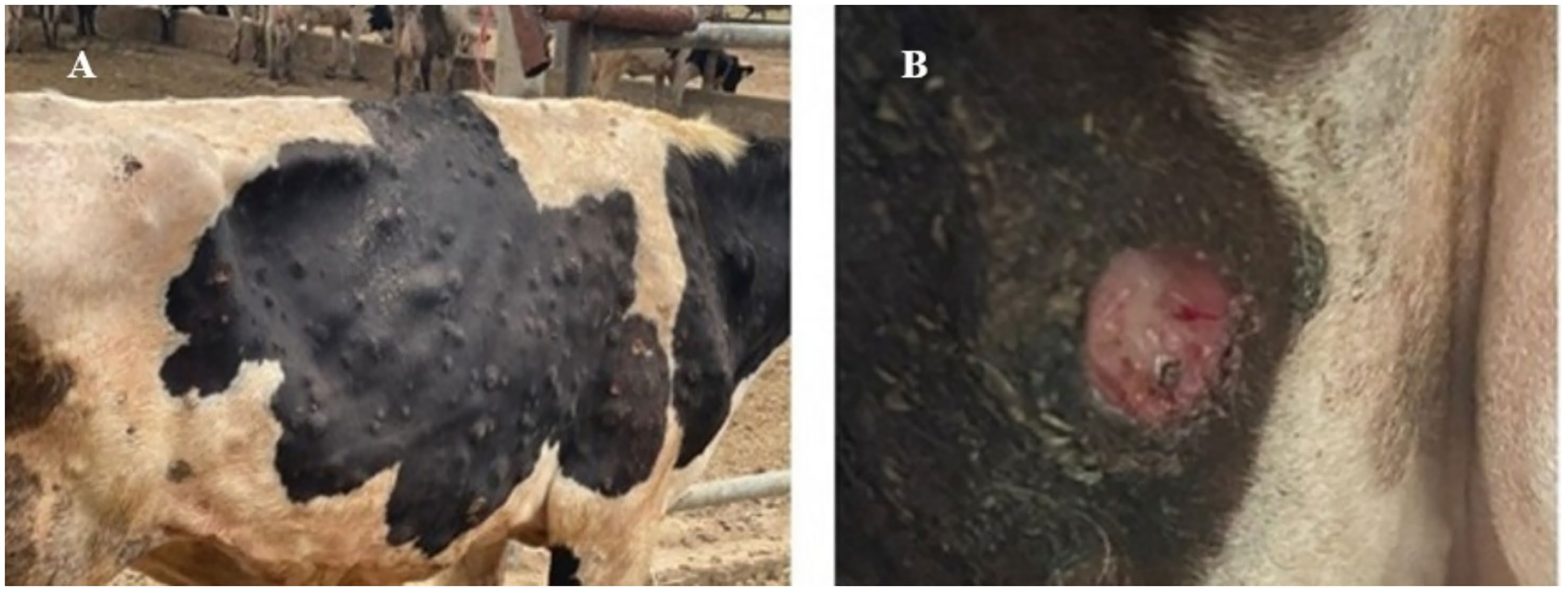
Clinical signs of cattle impacted by LSD. **(A)** Characteristic large firm skin nodules were distributed all over the body, **(B)** necrotic skin nodules and ulceration in the infected cow. Photo-graphed by Dr. Ahmed A. Elsayed.

Clinical examinations were performed for all suspected cases to check for LSD lesions. Distinctive, firm, circumscribed skin nodules measuring between 0.5 and 5 cm in diameter, covering every skin surface on the afflicted cattle, including the head, neck, trunk, perineum, udder, teats, subcutaneous tissue, and muscles, were the most important clinical symptoms (Figure 1A). The affected animals also had ulcerations and necrotic nodules (Figure 1B). Furthermore, several complications were displayed by the affected animals, including fever (40.5 ± 0.5 °C vs. 38.4 ± 0.13 °C) in the affected and control cows, respectively; elevated respiratory and heart rates (41.1 ± 1.3 vs. 26.9 ± 0.7 and 95.8 ± 2.5 vs. 50.7 ± 2.5) in the affected and control cows, respectively; depression; inappetence; decreased milk production; salivation; naso ocular discharge; pneumonia; corneal opacity; mastitis; and eventually the inability to stand up (recumbent animals).

### 3.2 Phylogenetic analysis

**Figure 2:**
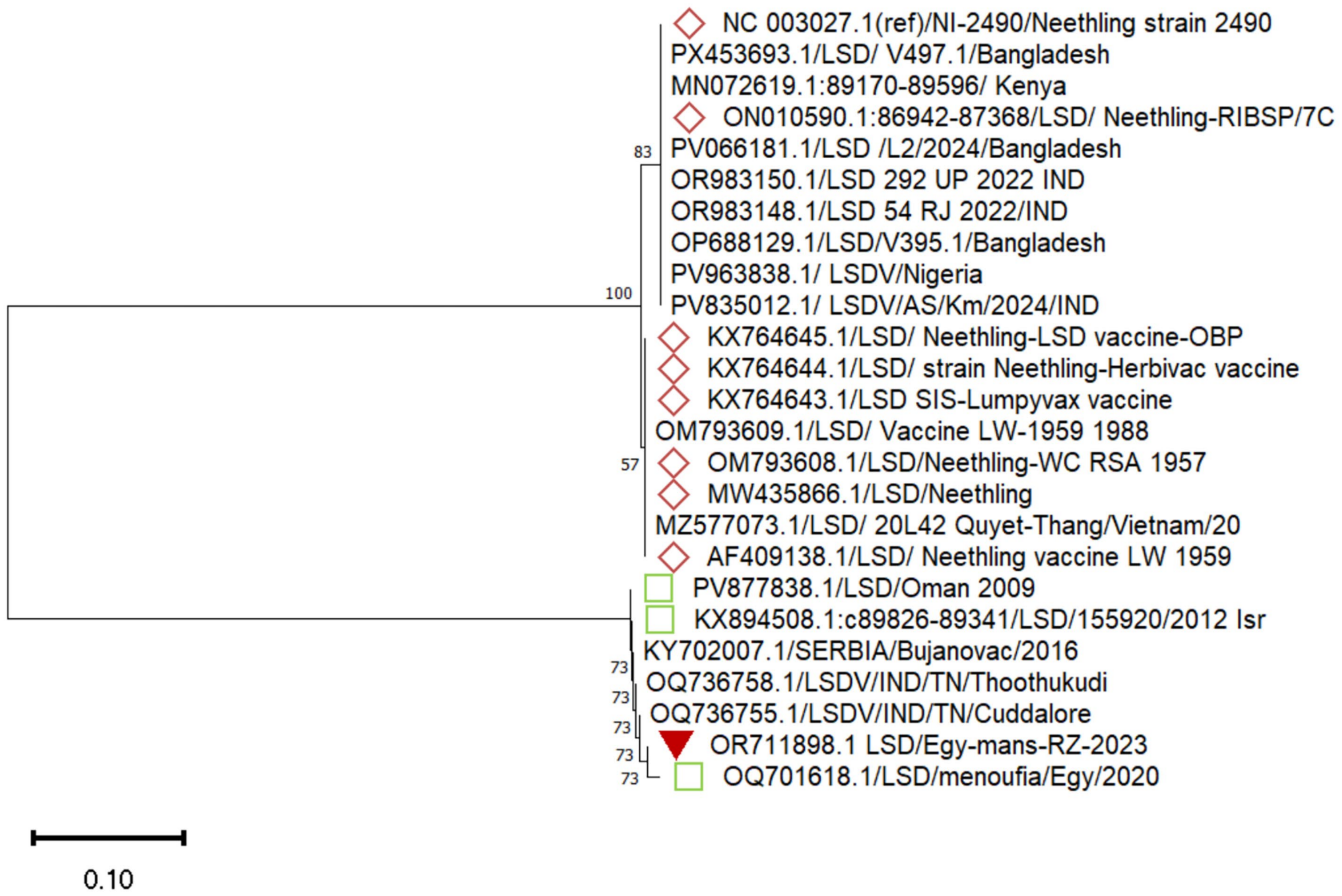
Phylogenetic analysis of LSDV based on partial ORF095 sequences. The tree was generated using Neighbor-Joining with 1,000 bootstrap replicates (values ≥50% shown). The isolate from this study (OR711898.1/Egy-mans-RZ-2023, red triangle) clusters with field strains from different countries and is distinct from Neethling vaccine strains (diamonds). Accession numbers are indicated; scale bar = 0.10 substitutions/site.

All the tested blood samples yielded positive PCR bands at 483 bp. One of them was sequenced, and the raw DNA sequence data were trimmed, aligned, and used to generate a consensus sequence, which was then compared with recent LSDV isolates reported in Egypt. The obtained sequence was submitted to GenBank under the accession number OR711898. Phylogenetic analysis revealed that our sample clustered with the 2020 Egyptian field strain, indicating genetic stability of this viral protein (Figure 2).

**Figure 3:**
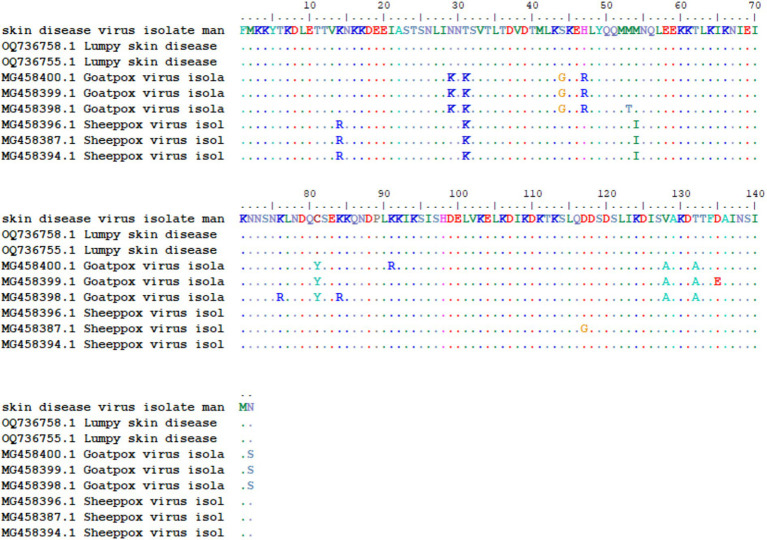
Multiple amino acid sequence alignment of the ORF095 protein of the LSDV isolate from this study compared with representative LSDV, goatpox, and sheeppox virus reference strains. Dots indicate identical residues, while letters denote amino acid substitutions. Sequence positions are numbered according to the reference LSDV sequence.

Notably, it did not cluster with the Neethling vaccine strain available in the Gen-Bank. A divergence analysis showing the genetic distances among the aligned sequences and multiple sequence alignments is illustrated in Figure 3.

### 3.3 Histopathological examination

**Figure 4:**
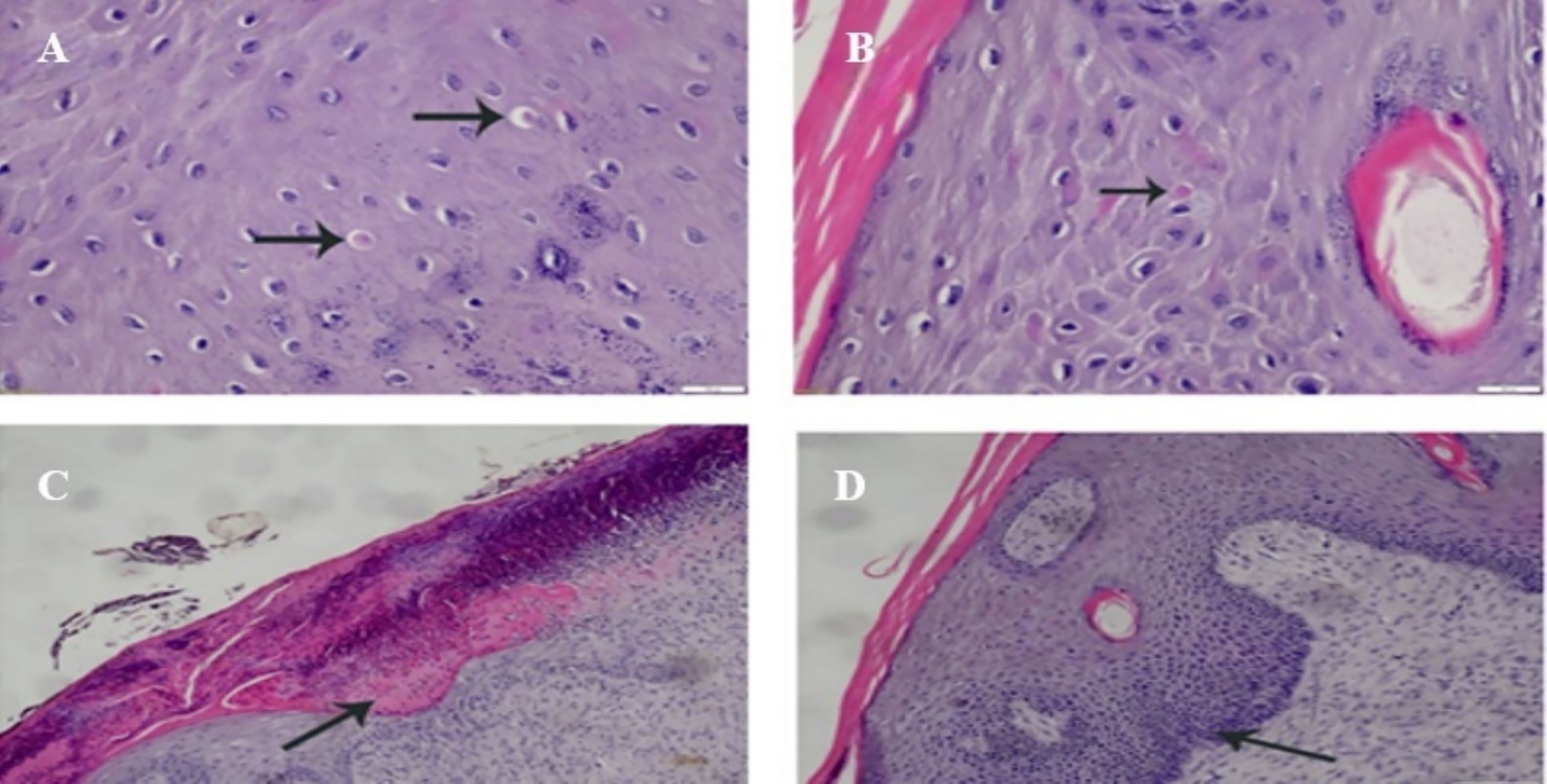
**(A)** Skin is displaying eosinophilic intracytoplasmic inclusion body (arrow) in the epi-dermal cell layer (HE, 400×). **(B)** Skin is displaying eosinophilic intracytoplasmic inclusion body (arrow) in the stratum spinosum of the epidermal cell layer (HE, 400×). **(C)** Necrosis (arrow) and scab formation in the overlying skin, with presence of basophilic bacterial colonies in the skin surface (HE, 100×). **(D)** Skin is displaying increased thickness of the epidermal cell layer and increase the proliferative if the basal cell layers (arrow) (HE, 100×).

Histological examination revealed eosinophilic intracytoplasmic inclusion bodies characteristic of LSD in the epidermal cell layers (Figures 4A,B). Ulceration of the skin, fibrinous exudate, and scab formation (Figure 4C). Hyperplastic proliferation and bacterial invasion are secondary changes due to the viral cytolytic effect (Figure 4D).

### 3.4 Biochemical findings

Compared to healthy cattle, LSD-infected cattle had significantly (*p* > 0.05) lower serum glucose, cholesterol, total protein, albumin, calcium, inorganic phosphorus, sodium, and potassium levels, while their serum levels of urea and creatinine, as well as their serum activity of AST, ALT, GGT, LDH, and CPK were higher (Table 2). When comparing the oxidative stress indicators of LSD-infected cattle to those of the control group, MDA significantly increased, whereas GSH, GPx, and catalase significantly decreased (Table 2). Regarding cytokine alterations, serum levels of IL4, IL6, TNF-*α*, IgA, IgM, IgG, *β* globulins, and *γ* globulins were significantly (*p* < 0.05) higher and α globulin values were significantly (*p* < 0.05) lower in LSD-infected calves than in the control group (Table 3).

**Table 2:** Distribution of SNPs, type of mutation in immune and antioxidant genes in healthy (*n* = 50) and diseased (*n* = 50) cows.

Gene	SNPs	Healthy *n* = 50	Diseased *n* = 50	Total *n* = 100	Chi square value *χ*^2^	*p*-value	Type of mutation	Amino acid number and type
*TLR7*	G59A	26/50	0/50	26/100	35.1	0.001	Non-synonymous	20 G to E
	A124G	0/50	44/50	44/100	78.5	0.001	Non-synonymous	42 T to A
	G264A	19/50	−/50	19/100	23.4	0.001	Synonymous	88 L
*TLR8*	C39T	34/50	−/50	34/100	51.5	0.001	Synonymous	13 C
	T246C	23/50	−/50	23/100	29.8	0.001	Synonymous	82 P
*TLR9*	T32C	0/50	39/50	39/100	63.9	0.001	Non-synonymous	11 V to A
	C218T	31/50	−/50	31/100	44.9	0.001	Non-synonymous	73 V to A
*SOD3*	G55C	0/50	37/50	37/100	58.7	0.001	Non-synonymous	19 V to L
	T134C	0/50	20/50	20/100	25	0.001	Non-synonymous	45 M to T
*CAT*	A114G	24/50	−/50	24/100	31.5	0.001	Synonymous	38 T
*GPX1*	A115C	30/50	−/50	30/100	42.8	0.001	Non-synonymous	39 N to H
	T160C	0/50	19/50	19/100	23.4	0.001	Non-synonymous	54 W to R
	C223G	40/50	−/50	40/100	66.6	0.001	Non-synonymous	75 Q to E

**Table 3:** Changes in serum biochemical, immunological and oxidant/antioxidant values in healthy (*N* = 50) and diseased cows (*N* = 50) (mean values ± SE).

Parameters	Healthy group	Diseased group	*p* values
Glucose (mg/dl)	107.6 ± 1.4	92.6 ± 1.4*	0.002
Cholesterol (mg/dl)	68 ± 0.5	52.6 ± 1.2*	0.001
Total protein (g/dl)	8.6 ± 0.05	7.4 ± 0.05*	0.001
Albumen (g/dl)	3.4 ± 0.05	2.8 ± 0.05*	0.002
Urea (mg/dl)	19 ± 0.5	31 ± 0.5*	0.001
Creatinine (mg/dl)	0.8 ± 0.04	1.3 ± 16.7*	0.004
AST (U/L)	74 ± 2	117.6 ± 1.4*	0.001
ALT (U/L)	40 ± 0.5	63.3 ± 0.8*	0.001
LDH (U/L)	40 ± 2.8	94.6 ± 1.4*	0.001
CPK (U/L)	2 ± 0.05	5.6 ± 0.3*	0.001
GGT (U/L)	5 ± 0.05	10 ± 0.1*	0.001
Calcium (mg/dl)	8.7 ± 0.2	7 ± 0.05*	0.004
Phosphorus (mg/dl)	4.6 ± 0.08	3.7 ± 0.1*	0.004
Sodium (mmol/l)	183 ± 1.1	158.6 ± 2*	0.001
Potassium (mmol/l)	2.4 ± 0.05	1.8 ± 0.07*	0.005
GSH (mg/dl)	44 ± 1.1	31.3 ± 0.8*	0.001
GPx (U/ml)	60 ± 0.5	41 ± 0.5*	0.004
Catalase (U/l)	36.3 ± 0.8	26.3 ± 0.8*	0.001
MDA (nmol/ml)	7.1 ± 0.1	13.5 ± 0.2*	0.001
IL 4 (pg/ml)	8.9 ± 0.1	18.2 ± 0.3*	0.001
IL 6 (pg/ml)	10.6 ± 0.2	19.7 ± 0.7*	0.001
TNFα (pg/ml)	60.6 ± 2.3	116 ± 1.1*	0.001
IgA (ng/ml)	88 ± 1.1	108 ± 0.5*	0.001
IgG (ng/ml)	339 ± 2	397 ± 2.5*	0.001
IgM (ug/ml)	101.6 ± 4.4	133.3 ± 28	0.322
α globulins (g/dl)	0.6 ± 0.05	0.3 ± 0.05*	0.02
β globulins (g/dl)	0.32 ± 0.01	0.47 ± 0.04*	0.04
γ globulins (g/dl)	0.3 ± 0.08	0. 7 ± 0.02*	0.01

*Values 0. AST, Aspartate aminotransferase; ALT, Alanine transaminase; LDH, Lactic dehydro-genase; CPK, Creatinine phosphokinase; GGT, Gamma glutamyl tranferase; GSH, Glutathione reduced; GPx, Glutathione peroxidase; MDA, Malondialdhyde; TAC, Total antioxidant capacity; IL4, Interlukin 1; IL 6, Interlukin 6; TNF-α, Tumer necrosis factor alpha; IgG, Immunoglobulin G; IgA, Immunoglobulin A; IgM, Immunoglobulin M.

### 3.5 Patterns for transcript levels of immune, and antioxidant indicators

**Figure 5:**
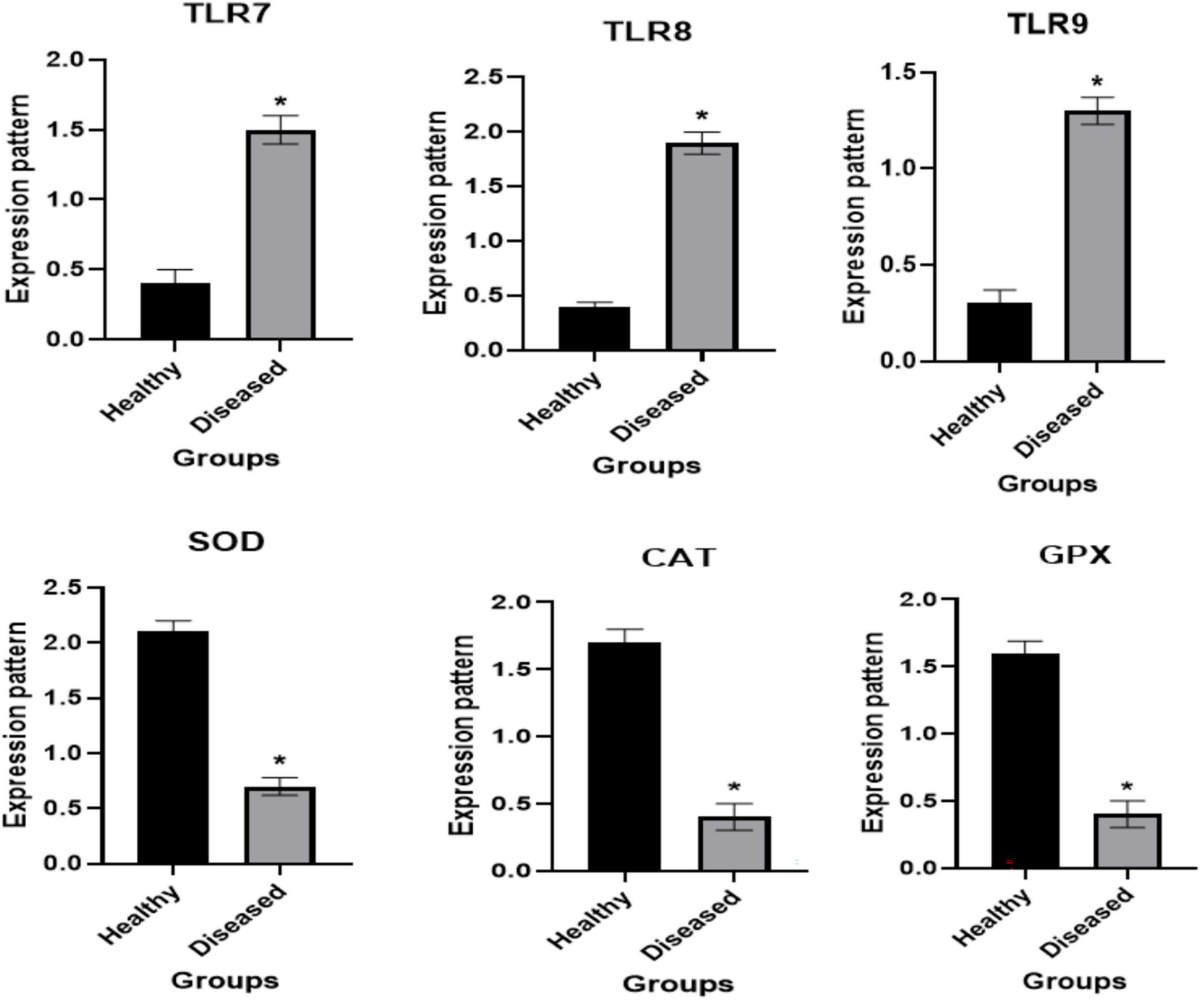
Different immune (TLR7, TLR8, and TLR9) and antioxidant (SOD3, CAT, and GPX1) genes transcript levels between normal and LSD affected cows. The symbol * denotes significance when *p* < 0.05.

Figure 5 shows the transcript profiles of the evaluated immune and antioxidant genes. The expression levels of TLR7, TLR8, and TLR9 were significantly upregulated in lumpy skin-affected cattle compared with those in resistant animals, whereas the antioxidant genes SOD3, CAT, and GPX1 were markedly reduced in affected individuals.

### 3.6 SNPs of immune and antioxidant genes

The DNA sequence verdicts of healthy and affected cows revealed differences in the SNPs in the amplified DNA bases related to lumpy skin disease incidence for TLR7 (305-bp), TLR8 (338-bp), TLR9 (385-bp), SOD3 (300-bp), CAT (344-bp), and GPX1 (343-bp) genes; submitted to GenBank with accession numbers PZ196058-PZ196063. All the discovered SNPs were validated using the DNA sequence differences between immune and antioxidant markers investigated in the cows and the reference gene sequences obtained from GenBank.

Table 2 summarizes the SNPs identified in six key immune and antioxidant genes (TLR7, TLR8, TLR9, SOD3, CAT, and GPX1) in a cohort of 70 cattle, equally divided between healthy (*n* = 50) and lumpy skin disease (LSD)-infected (*n* = 50) animals. Several SNPs were detected with a notable pattern of association between specific mutations and infection status. In TLR7, the G59A mutation (resulting in amino acid substitution G20E) was found exclusively in healthy animals (18/35), whereas the A124G mutation (T42A) was detected exclusively in infected cattle (31/35), suggesting a potential role in disease susceptibility. Other SNPs in TLR7 and TLR8 (e.g., G264A, C39T, and T246C) are synonymous and may not alter protein function directly. Non-synonymous mutations in TLR9 (T32C and C218T) lead to amino acid changes at positions 11 (valine to alanine) and 73 (valine to alanine), respectively. Similarly, SOD3 exhibited two non-synonymous SNPs (G55C and T134C), resulting in amino acid changes at positions 19 and 45, whereas CAT and GPX1 also harbored SNPs with potential functional consequences, such as A115C (asparagine to histidine) and T160C (tryptophan to arginine). These findings highlight specific polymorphisms that may influence individual variations in immune or oxidative stress responses during LSD infection, offering candidate variants for LSD status. The non-synonymous SNPs associated with LSD-affected animals were A124G in TLR7, T32C in TLR8, G55C in TLR9, T134C in SOD, and T160C in GPX1.

Significant differences were detected in the frequencies of all examined SNPs between LSD and healthy cows (*p* < 0.005). Chi-square analysis was carried out to compare the distribution of all identified SNPs in all genes between LSD and healthy cows. The total Chi-square value showed significant variation among the identified SNPs in all genes between the resistant and affected animals (*p* < 0.05; Table 2).

The discriminant analysis for the correlation between health status and gene types is shown in Table 4. The organizational ramifications demonstrated that the model correctly identified either healthy or diseased cows in 100% of situations. According to these findings, the SNP markers utilized in the model had a high degree of discriminatory power and could be helpful as candidate variants associated with LSD status.

**Table 4:** Discriminant analysis for classification of type of genes and healthy status of examined cows.

		Predicted group membership		Total
		Healthy	Diseases	
Count	Healthy	50	0	100
	Diseased	0	50	100
%	Healthy	50	0.0	100.0
	Diseased	0.0	50	100.0

## 4 Discussion

Lumpy skin disease (LSD) is recognized as a transboundary animal disease owing to its profound impact on international trade and food security, as well as its potential to spread rapidly across national borders (37). In the present study, the integration of clinical assessment, molecular confirmation, and haematobiochemical analysis demonstrated clear distinctions between LSD-affected and control cattle, thereby validating the study hypothesis and confirming that LSD infection is associated with significant systemic, metabolic, and inflammatory alterations that can be reliably detected using these diagnostic approaches.

Genetic variation within livestock populations plays a pivotal role in modulating individual immune responses to infectious agents (38–40). Understanding the genetic factors underlying the different susceptibilities offers valuable insights for the development of targeted therapeutic strategies and informed breeding programs (41). Natural outbreaks of LSD in endemic regions present a unique opportunity to investigate the genetic basis of disease resistance/susceptibility under real-world, relatively uniform environmental conditions. Importantly, immune responses observed in natural bovine hosts *in vivo* often differ markedly from those studied in non-native or *in vitro* models, emphasizing the necessity of evaluating host–pathogen interactions in biologically relevant systems (42).

In this study, LSDV was identified based on clinical symptoms and confirmed using PCR results. The diseased cows had fever, anorexia, emaciation, decreased milk production, skin nodules, lacrimation, nasal discharge, and edema in the dewlap and limbs according to a clinical examination. The aforementioned clinical indicators are detailed in (43–46).

According to (47), PCR is the most effective method for quickly identifying and locating the causative agent of the viral outbreak under investigation. All blood samples tested positive by conventional PCR targeting ORF 95. ORF genes in the genome of LSDV are conserved and have served as a target to perform PCR for virus diagnosis with high accuracy, as in the reports of previous researches (48, 49), which used ORF 95, 103, and GPCR genes to detect the virus with high accuracy. In contrast, another researcher used the LSD044 region in the LSDV genome to generate primers and probes to detect LSDV by real-time PCR (50).

ORF 95 amino acid sequence alignment with sheep and goat pox viruses showed a characteristic difference, indicating that it has the potential to differentiate between the members of the capri pox virus genus. Although this hypothesis is likely to be proven by our study, it is still to be tested using a larger panel of sequences and a wet-lab platform.

The LSDV ORF 95 gene was detected in all blood samples tested from a herd of cattle vaccinated with a Neethling-strain-based live attenuated vaccine. However, this sequence showed dissimilarity to the Neethling vaccine ORF 95 gene (51). This finding carries a great risk of circulation of field viruses that vary from vaccinal strains or encounter mutations and recombination events, as reported earlier (52), and it is well established that such genetic variations are better detected and evaluated by full genome sequencing.

The spot lesion of LSD is the intracytoplasmic inclusion body, which is usually large and in the epidermal cell layer known as ‘sheep pox’ cells (SPCs) or ‘cellules claveleuses’ of Borrel in any of the skin biopsies (53). The cytolytic effect of the virus and rubbing of the lesion against hard objects allows ulceration on the skin surface with complications due to secondary bacterial infection and attempts of the skin to heal by hyperplastic alterations in the basal cell layers to renew the loss the epidermal cell layers. These histopathological findings have been reported previously (44, 54).

According to the electrophoretic pattern, the infected group’s levels of total immunoglobulin, particularly the gamma globulin fraction, significantly increased, whereas total protein and albumin significantly decreased. These findings may be explained by damaged liver parenchyma, increased catabolic rate, and decreased synthesis. Some studies have explained these outcomes by blaming the reduced food intake during anorexia. This also explains why serum glucose levels in animals with infection are declining. In contrast, elevated gamma globulin levels, particularly IgG immunoglobulin levels, are mostly an immunological reaction to infection. These results are consistent with the findings of previous studies (43, 45). According to Sanz et al. (54), hypocholesterolemia in animals infected with LSDV may be caused by the reduced absorption of cholesterol as a result of vasculitis in the small intestine. Hypocholesterolemia may arise from increased fecal bile acid excretion, increased cholesterol transit in the large intestine, and an improved rate of macrophage-specific reverse cholesterol transport in stressful illness conditions (55).

In relation to the hepatic and renal biochemical investigations, the activity of AST, ALT, LDH, GGT, and CPK in infected cows was significantly higher than that of healthy cows. According to previous research (56), the impact of LSDV infection on hepatic enzyme activity may be related to the overall tissue breakdown caused by the virus or secondary invaders, as well as the hepatocellular damage caused by different agents. Therefore, muscle injury resulting from sick pathophysiology is responsible for altered hepatic enzyme production in animals infected with LSD (57). Viral exposure also affects renal function, as evidenced by the large increases in urea and creatinine levels. These changes may have been caused by degenerative changes in the kidneys and liver, suggesting a decrease in the glomerular filtration rate (58). These outcomes concur with those reported in earlier studies (59, 60). Conversely, calves that were naturally infected with LSDV had low creatinine levels (61). The time of sampling, methods employed for the blood biochemical analysis, individual variations, age, breed, sex, nutrition, heat stress, lactation, and pregnancy phase are some possible factors for this divergence.

In general, lower synthesis, higher catabolic rate, damaged liver parenchyma, decreased food consumption, or impaired absorption of these elements could be the cause of the marked decrease in mineral concentrations (calcium, inorganic phosphorus, sodium, and potassium). These results concur with those published previously (62).

Studies characterizing blood antioxidant and lipid peroxidation status in cattle skin disorders are scarce. In LSDV-infected cattle, the current study found significantly lower serum GSH, GPx, and CAT levels and elevated MDA levels. These findings were linked to increased oxidative stress caused by the production of free radicals and lipid peroxidation as a result of the low running of the blood antioxidant reserves, which leads to tissue damage (63). Our findings are consistent with those published previously (62, 64).

According to a previous study (64), oxidative stress increases the synthesis of oxidants, such as MDA, which might affect the release of proinflammatory mediators, such as cytokines. These mediators are crucial for inflammation in several skin conditions. According to our study, infected cattle had higher than normal levels of IL-4, IL-6, and TNF-*α*. This could be related to the stimulation of macrophages and lymphocytes, which causes them to release cytokines that cause inflammation in various organs during the viremic stage of the disease. Our results are comparable to those of other studies (45, 62).

By measuring the mRNA levels of immune (TLR7, TLR8, and TLR9) and antioxidant (SOD3, CAT, and GPX) genes, we examined the changes in the immune and antioxidant states in lumpy skin-affected cows compared to healthy ones. Gene expression levels were considerably higher in affected cows than in resistant cows for TLR7, TLR8, and TLR9. The SOD3, CAT, and GPX genes were expressed at substantially lower levels in the affected cows.

This is the first study to analyze the transcript levels of immune and antioxidant indicators linked to the hazard of lumpy skin disease. Five non-synonymous SNPs in TLR genes and antioxidant-related genes were found to be significantly associated with infected rather than healthy cattle recruited in this study. To the best of our knowledge, this study is the first to propose ‘A124G in TLR7, T32C in TLR8, G55C in TLR9, T134C in SOD, and T160C in GPX1’ as potential biomarkers associated with infection or susceptibility to LSD in Holstein cattle. Several of the identified SNPs are non-synonymous and result in amino acid substitutions that may influence protein structure or function (65). Variants within TLR7, TLR8, and TLR9 occur in regions known to be involved in pathogen recognition and downstream signaling, suggesting potential effects on antiviral immune responses. Likewise, non-synonymous SNPs in SOD3, CAT, and GPX1 may alter enzymatic activity or regulate oxidative stress during infection.

Consequently, qualitative and quantitative differences in the expression of the investigated genes precede the development of lumpy bovine skin disease. According to (66), the expression profiling of Th1 and Th2 cytokines revealed significant differences between healthy and lumpy skin-affected Vrindavani cattle, except for IL10. The expression of IL2, GMCSF, and IL6 was upregulated in healthy animals, whereas that of INFG, IL4 and IL10 was upregulated in LSD-affected animals. The highest abundance was observed for IL2 transcripts in healthy animals among all assessed cytokines, with a log2fold change of 1.61 as compared to their affected counterparts.

Several studies have reported the essential role of IL6 in mounting an effective immune response against viral pathogens (67), especially during early infection (68). IL6 is a pleiotropic molecule that is produced from multiple cell lineages and is involved in stimulating cascades related to the JAK/STAT3 pathway, which subsequently leads to the activation of several genes associated with immunomodulation involving both pro- and anti-inflammatory chemokines (69–71). In LSD-affected animals, increased production of INFγ could have been responsible for the exaggerated symptoms resulting from increased necrosis and tissue damage. These changes are associated with altered liver metabolism due to an inflammatory state or hepatic damage due to viremia (72). These findings imply that variations in gene expression in the immune and antioxidant pathways may affect the capacity of cows to withstand lumpy skin diseases.

The immune (TLR7, TLR8, and TLR9) and antioxidant (SOD3, CAT, and GPX) gene sequences of lumpy skin-affected and healthy Holstein dairy cows were characterized in this study. The findings showed that SNPs in both categories varied; submitted to GenBank with accession numbers PZ196058-PZ196063. It is important to emphasize that the polymorphisms found and made available in this context provide additional data for the evaluated indicators compared to the corresponding datasets acquired from GenBank. Genetic polymorphisms in bovine immune-related genes are recognized as major contributors to the variability in host responses to infectious diseases (72). Previous studies have demonstrated extensive SNP diversity in genes encoding toll-like receptors (TLRs) and antioxidant enzymes, which play central roles in pathogen recognition, immune activation, and oxidative stress regulation during viral infections in cattle (65, 73).

Polymorphisms in TLR7, TLR8, and TLR9 have been associated with altered antiviral signaling, cytokine production, and susceptibility to viral diseases in cattle. These receptors are responsible for sensing viral nucleic acids and initiating innate immune responses, making them biologically plausible candidates for influencing disease outcome (72). The non-synonymous SNPs identified in the present study overlapped with gene regions previously reported to be variable in cattle, supporting their potential relevance to antiviral immunity.

Similarly, genetic variations in antioxidant-related genes, including SOD3, CAT, and GPX1, have been linked to differences in oxidative stress tolerance and inflammatory responses in cattle. SNPs in these genes may influence the ability of animals to control infection-induced oxidative damage, thereby modulating disease severity (19). Immune-related SNPs have been widely studied in relation to mastitis, respiratory infections, and parasitic diseases (29). Host genetic data specific to lumpy skin disease remain scarce, with most studies focusing on viral rather than host genomics. In this context, the present findings extend the existing bovine immunogenetic background by identifying host immune and antioxidant gene variants associated with the LSD status. Importantly, these SNPs represent pre-existing host genetic variations rather than infection-induced changes, and their differential distribution between healthy and infected animals suggests a potential association with susceptibility or resistance, although causal relationships require validation in larger independent populations.

In mammals, Toll-like receptor genes (TLRs) facilitate host recognition of pathogen-associated molecular patterns (PAMPs), eliciting host innate immune responses (74, 75) aimed at suppressing invading bacteria, viruses, protozoa, and fungi. Six gene family members (TLR1, TLR2, TLR4, TLR5, TLR6, and TLR9) are known to recognize microbial (bacteria, fungi, protozoa) and/or synthetic ligands, and five (TLR3, TLR4, TLR7–TLR8, TLR9) are known to recognize viral components (76, 77). Ten different TLRs (TLR1–TLR10) have been identified in cattle. Among these, TLR2, TLR4, TLR6, and TLR9 have been found to play a role in innate immunity (78, 79). Antioxidants provide protection by removing ROS from the environment, limiting their synthesis, or securing transition metals that are used to create free radicals (80). These processes include enzymatic and non-enzymatic antioxidant defenses such as catalase (CAT) and superoxide dismutase (SOD), which are endogenous antioxidant indicators (81).

A thorough understanding of the genetic mechanisms driving the optimal immune response against the pathogenic agent helps in designing meticulous disease control programs and developing effective vaccines (82). The natural outbreak of LSD in animals and assessment of the immune response provides a better opportunity to gain deeper insights into the genetic basis of disease resilience in natural hosts (bovines for LSD) under near-uniform environmental conditions (83). Elucidating the expression profiles of immune and antioxidant genes provides an opportunity to better understand the immune function and response of animals against infectious pathogens. Innate immunity, although non-specific, forms the first line of defense in biological organisms and involves the complex functioning of many cell types (11). It helps in stimulating cascades to effectively mount the immune response against infectious pathogens. These cells are characteristic of inflammatory responses and are involved in stimulating the release of cytokines, chemokines, and other immunomodulatory molecules that are directly related to an effective immune response.

The present study showed some limitations that should be considered in future studies. First, the current investigation was carried out using a small sample size and animals from a single private farm. Therefore, future studies involving larger populations across multiple farms and geographical regions are warranted to validate and extend the applicability of our results. Second, Although PCR was used as a reliable method for detecting active LSDV infection, the absence of serological testing (e.g., ELISA or virus neutralization assays) may limit the assessment of prior exposure and humoral immune status. Future studies incorporating both molecular and serological approaches are recommended for comprehensive disease evaluation. Third, the phylogenetic analysis in this study was based on partial sequencing of a single gene (ORF95) from samples collected from a single farm. However, recent work has demonstrated that additional genomic regions, such as ORF134, are valuable for detecting recombinant LSDV strains and providing a more comprehensive understanding of viral evolution (84). Therefore, the findings provide only preliminary insights into viral genetic relatedness and do not allow comprehensive conclusions regarding viral evolution or wider epidemiological patterns. Future studies involving whole-genome sequencing and samples from multiple locations are recommended. Fourth, a limited number of samples were subjected to molecular and phylogenetic analysis due to sample selection criteria and sequencing constraints. Although sufficient for identifying circulating strains, larger sample sizes would provide a more comprehensive assessment of viral diversity and epidemiology. Fifth, only a limited number of genes related to immunity and antioxidants were examined. In addition, the identification of individual SNPs in the present study should be interpreted cautiously, as the detection of a single polymorphism does not necessarily imply causal susceptibility to lumpy skin disease. Disease resistance and susceptibility are complex traits influenced by polygenic interactions, environmental conditions, and herd management factors. Therefore, the reported SNPs should be considered candidate association markers rather than definitive predictors of disease outcome. Therefore, a wide range of factors must be considered in subsequent studies. Sixth, this study was conducted on a single cattle breed, which may limit the generalizability of the findings to other breeds or production systems. Future investigations involving larger sample sizes, multiple herds, and geographically diverse populations are required to validate these polymorphisms and confirm their biological relevance. Seventh, although functional assays were not performed, in silico prediction tools such as SIFT, PolyPhen-2, and PROVEAN can be applied in future studies to evaluate whether these variants are likely to be deleterious or functionally impactful. Therefore, the functional consequences of these SNPs should be considered putative and warrant further validation through bio-informatics and experimental approaches. Finally, A limitation of the present study is that the phylogenetic tree was constructed without inclusion of an outgroup. Consequently, the analysis is restricted in its ability to infer the directionality of evolutionary relationships among the analyzed strains. This limitation is primarily related to the short and relatively conserved nature of the ORF95 gene fragment used, which complicates the selection of an appropriate and informative outgroup. Future studies should consider incorporating suitable outgroup sequences, ideally based on longer genomic regions or whole-genome data, to enhance phylogenetic resolution and enable more robust evolutionary interpretations.

## 5 Conclusion

Overall, this study provides novel molecular and genetic evidence linking host immune–antioxidant gene variation to differential susceptibility to lumpy skin disease viruses in cattle. Our findings underscore the relevance of SNPs in immune and antioxidant genes as candidate variants associated with LSD status, rather than definitive predictive markers. Validation in independent cohorts and prospective studies is required before their application in breeding or disease prediction programs. The observed variability in these genes may serve as surrogate biomarkers to predict disease susceptibility in cattle. Furthermore, the distinct expression patterns between resistant and susceptible animals provide a valuable reference for monitoring host responses to LSDV. The integration of genomic, immunological, and pathological indicators provides a comprehensive diagnostic framework for improving early detection and control of lumpy skin disease in cattle. Future studies should validate these candidate genetic markers in larger and genetically diverse cattle populations, integrate whole-genome and functional genomics approaches, and evaluate gene environment interactions that influence LSDV susceptibility. Additionally, longitudinal studies linking genetic profiles with vaccine responsiveness, production traits, and field resistance are warranted to support the practical implementation of marker assisted genomic selection programs for sustainable LSDV control.

## Data Availability

The original contributions presented in the study are included in the article/supplementary material, further inquiries can be directed to the corresponding authors.

## Footnotes

1
https://www.ncbi.nlm.nih.gov/genbank/
